# Shotgun metagenomic and phenotypic characterization of indigenous lactic acid bacteria from raw milk artisanal cheeses: metagenomic functional insight and starter culture traits

**DOI:** 10.3389/fmicb.2026.1820264

**Published:** 2026-06-04

**Authors:** James N. Nelon, Sama S. Eltaher, Ahmed G. Abdelhamid

**Affiliations:** 1 Department of Food Science and Human Nutrition, Michigan State University, East Lansing, MI, United States; 2 Department of Microbiology, Genetics, and Immunology, Michigan State University, East Lansing, MI, United States

**Keywords:** artisanal cheeses, cheese microbiota, fermentation, lactic acid bacteria, shotgun metagenomics, starter cultures

## Abstract

The diversity of commercial starter cultures of lactic acid bacteria (LAB) used in fermented dairy products is limited. This has created strong demand to discover novel starter culture strains to develop unique products with appealing sensory characteristics. The current study used an integrated shotgun metagenomic and culture-based pipeline to (a) define taxonomic composition and functional potential of selected artisanal raw milk cheese microbiomes and (b) isolate and evaluate native LAB strains as potential starter cultures. Five artisanal cheeses (brie, bleu, plain gouda, mustard seed gouda, and nettle gouda) were analyzed. Shotgun metagenomics profiled the cheese microbiomes and revealed a high abundance of *Lactococcus cremoris* and *Lactococcus lactis* in gouda cheeses, whereas brie cheese contained high abundances of *L. lactis* and *Streptococcus thermophilus*. Functional profiling of metagenome-assembled genomes recovered from cheese microbiomes identified abundant pathways linked to carbon utilization, energy metabolism, and organic nitrogen metabolism. In parallel, 12 LAB isolates were recovered from all cheeses, of which five strains were classified taxonomically as *L. lactis* using whole genome sequencing. These five *L. lactis* strains displayed desirable milk and cream fermentation properties, achieving coagulation within 6 h, with final pH values of 4.5. The resulting fermented products contained 2.9%−4.2% protein content, displayed a relative increase in long-chain fatty acids, and a relative decrease in short-chain fatty acids compared to unfermented controls. The current study links cheese metagenome functional potential to dairy adaptation and identifies indigenous *L. lactis* strains as promising candidates for novel starter cultures in fermented dairy products.

## 1 Introduction

In the dairy industry, commercial starter cultures used in the production of fermented dairy products such as yogurts, cheeses, and sour cream products are comprised of a mixture of various lactic acid bacteria (LAB) (Fox et al., 2017). LAB are noteworthy in dairy food production for their ability to lower the pH of food products, inhibit spoilage, and contribute unique sensory characteristics to foods, while tolerating stress-inducing conditions like low pH and oxygen-depleted environments (Anumudu et al., 2024). *Lactococcus lactis* (*L. lactis*) stands out in dairy production due to its genes involved in the metabolism of lactose and a diverse set of milk proteins (Cavanagh et al., 2015). The use of LAB starter cultures is notable for their ability to produce products with consistent, safe, and desirable qualities, making them highly regarded by industries seeking to create large-scale fermented dairy products (Siddiqui et al., 2023).

When specifically considering dairy production processes, it is well known that pasteurization of milk products is particularly effective at eliminating bacteria originating from both the raw milk itself and the production environment (Rabbani et al., 2025) such as dairy equipment, ripening surfaces, and from traditional materials such as wooden vats. However, pasteurization processes eliminate both pathogenic and non-pathogenic bacteria in raw milk, rendering the product free of the beneficial bacteria from the raw milk microbiota (Asaithambi et al., 2021). This then requires the use of commercial starter cultures to produce cheeses and other fermented dairy products (Coelho et al., 2022). Notably, some small-scale, artisanal farms and cheesemakers use raw, unpasteurized dairy products in cheese production (Stefanou et al., 2022). Thus, these cheeses can serve as a reservoir of LAB that can be used as novel starter cultures, contributing to unique sensory and nutritional benefits (Sant'Anna et al., 2019).

Shotgun metagenomics is driving the field by enabling the identification of microbial communities in fermented foods and the study of their interactions without labor-intensive laboratory techniques (Yasir et al., 2022). It is increasingly used in investigating microbial ecosystems, building microbial consortia for food fermentation, and identifying metabolic pathways that modulate microbial interactions in food (Carlino et al., 2024). Given that LAB often exist in communities, metagenomics would be a useful approach for studying their interaction characteristics and their community-dependent properties (Nam et al., 2023).

For identifying novel LAB isolates for use as starter cultures in fermented dairy products, it is vital to ensure that starter cultures produce consistently fermented, safe, and organoleptically stable products (Bai et al., 2024). Thus, novel starter cultures must be tested individually for their ability to coagulate and acidify dairy products. To obtain a comprehensive assessment of the novel LAB isolates, nutritional analyses (e.g., protein content, fatty acid profile, moisture content) should be performed in the resulting fermented product to verify that such isolates are suitable for use in food products (Ikele et al., 2024). Therefore, this study aims to (i) apply shotgun metagenomics to characterize the microbial composition and functional potential of selected artisanal cheeses, and (ii) isolate and characterize novel LAB strains and test their use as potential starter cultures.

## 2 Materials and methods

**Figure 1:**
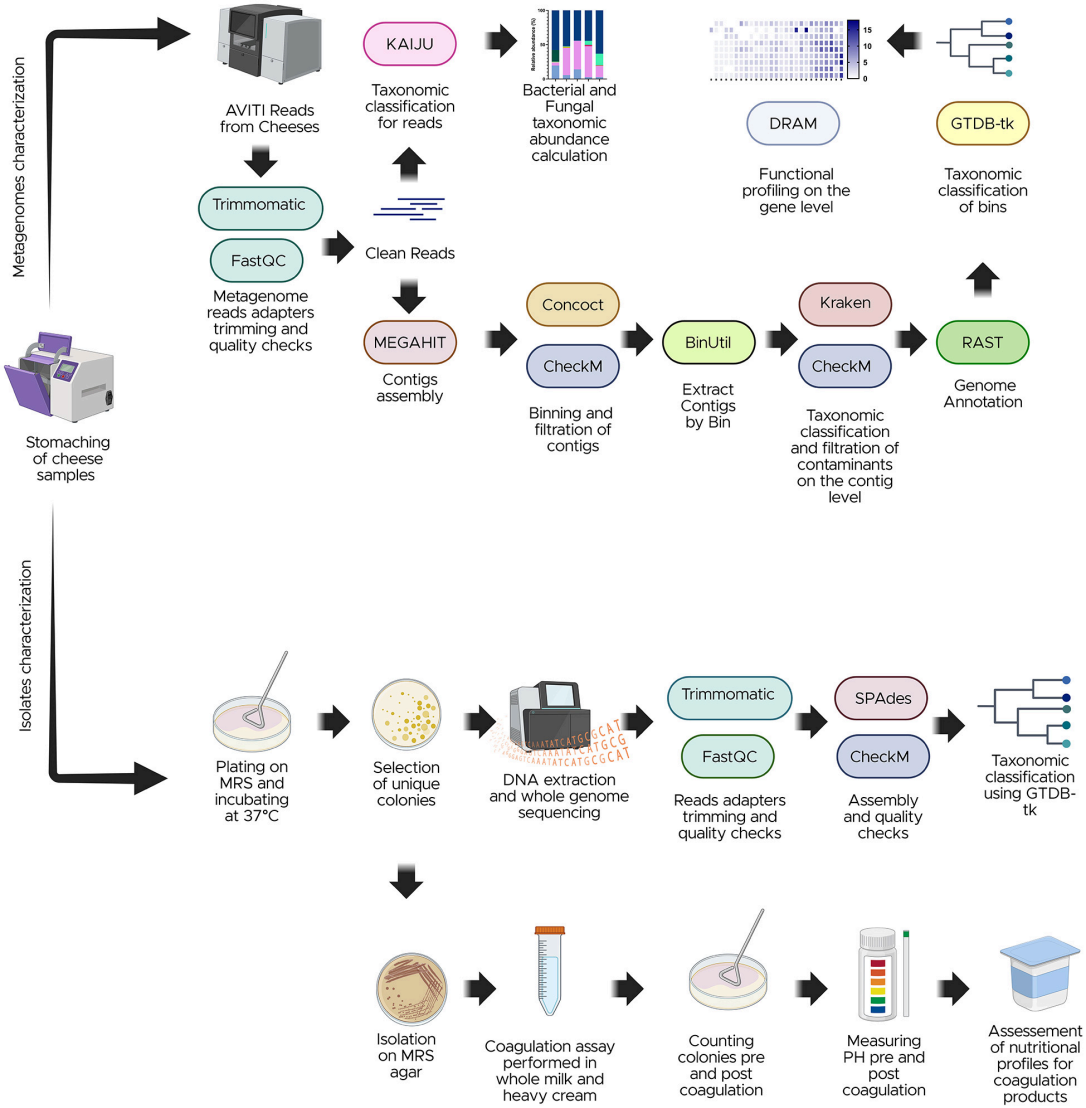
A schematic illustration of the study workflow. The study included two parallel analyses: (i) Shotgun metagenomic analysis of the cheese metagenomes and (ii) wet lab experiments on isolation of selected lactic acid bacteria, their whole genome sequencing, and fermentation of milk and cream. (created in BioRender.com).

A schematic diagram for the methodology workflow is illustrated in Figure 1. The current study implemented two approaches. Shotgun metagenomics was used to characterize the microbial composition of cheeses and depict the functional properties of some genomes derived from the cheese metagenomes. In addition, isolation and characterization of the isolated LAB strains and their potential in producing fermented milk and cream were assessed.

### 2.1 Isolation of lactic acid bacteria (LAB) from artisanal cheeses

Five artisanal, raw milk cheeses, comprising three varieties, were purchased from a local Farmers market in Okemos, Michigan, United States of America, in October of 2024. Varieties included Brie (*n* = 1), Bleu (*n* = 1), and Gouda (*n* = 3). Among the cheeses analyzed, only the Brie was manufactured using a blended milk (raw and pasteurized milk) inoculated with starter cultures by the producer while all other cheeses were produced and fermented solely with native LAB. For sampling, equivalent portions of each cheese (5 g) were placed into stomaching bags (Fisher Scientific, Pittsburgh, PA, USA) along with 20 mL of a sterile aqueous solution of 2% sodium citrate (Fisher Scientific) pre-heated to 60 °C. Samples were homogenized using a food stomacher (Seward, Worthing, West Sussex, UK) on the highest setting for 60 s. The homogenate was prepared in 1x Phosphate-Buffered Saline (PBS) (Fisher Scientific) and diluted tenfold serially. Aliquots of the dilutions (100 μL each) were plated onto de Man, Rogosa, and Sharpe (MRS) agar (Oxoid, Hampshire, UK). Inoculated plates were incubated aerobically in a standard benchtop incubator (Precision Scientific, USA) at 37 °C for 36 h. After incubation, plates were examined, and colonies with unique morphologies were selected, streaked onto MRS agar, and incubated at 37 °C for 36 h. Frozen stocks of the isolated colonies were prepared by inoculation into 1 mL of a 1:1 MRS-glycerol (Thermo Fisher Scientific, Waltham, MA, USA) solution. Stocks of isolates were kept at −80 °C for further use.

### 2.2 Metagenomic sequencing and analysis of cheeses

Total DNA was extracted from the homogenates of each of the five artisanal cheese varieties using the DNeasy Metagenomic Food DNA Extraction Kit (Qiagen, Germantown, MD) according to the manufacturer's instructions. Libraries were prepared using a DNA library preparation kit (Quantbio SparQ DNA Library Prep, Quantabio, Beverly, MA, USA) and indexed with dual index kit (IDT xGen 10nt Unique Dual-Index adapters, IDT, Coralville, IA, USA) following the manufacturer's recommendations. Completed libraries were checked for quality and quantified using two dsDNA quantification kits (Biotium AccuGreen High Sensitivity dsDNA and Agilent 4200 TapeStation High Sensitivity D1000 assay kit). The libraries were normalized and pooled in equimolar amounts. The shared pool was sequenced using an AVITI Cloudbreak Freestyle 2 × 150 High Output Kit (Element Biosciences, San Diego, CA). Base calling was done by AVITIOS 2.6.2 (Element Biosciences), and the output was demultiplexed and converted to FastQ format using bases2fastq v2.0.0 (Element Biosciences).

The sequenced metagenomic reads were uploaded to the KBase platform for metagenomic analysis (Arkin et al., 2018) as illustrated in Figure 1. Quality checks were performed with FastQC (v0.12.1) (Andrews, 2010), and adaptors were trimmed with Trimmomatic (v.36) (Bolger et al., 2014). Afterward, cleaned reads were screened with KAIJU (v1.9.0) (Menzel et al., 2016) using fungal and bacterial databases (RefSeq Genomes) to determine relative taxonomic abundances within the analyzed cheeses. Kaiju was run in Greedy mode with default parameters, including a minimum match length of 11 amino acids, minimum match score of 65, maximum of 3 mismatches, and E-value cutoff of 0.01. The taxonomic abundance figures were generated using GraphPad Prism (version 10.6.1). Following microbial composition analysis, the fastq files were assembled using MEGAHIT (v1.2.9) (Li et al., 2015), and contigs were then binned using CONCOCT (v1.1) (Alneberg et al., 2014), and filtered by CheckM (v1.0.18) (Parks et al., 2015). The inclusion criteria for extracted genomic bins were completeness greater than 90% and contamination less than 10%. For bins with contamination more than 10%, the binutil (v1.0.2) tool was used to extract bins, followed by taxonomic classification of contigs with kraken2 (v2.1.3) (Wood et al., 2019). The contaminant contigs were then filtered out, and completeness of the binned contigs was confirmed using CheckM. Assembly and quality metrics of MAGs are shown in Supplementary file 1. For the taxonomic classification and functional analyses of the binned contigs, RAST was used for functional annotation (v1.073) (Aziz et al., 2008), followed by executing the genome taxonomy database-toolkit GTDB-Tk (v2.3.2) (Chaumeil et al., 2019) for microbial taxonomic classification. Additionally, DRAM (v0.1.2) (Shaffer et al., 2020) was used to generate functional profiles for each extracted genomic bin.

### 2.3 Phenotypic approaches for screening the starter Lactic acid bacteria strains

#### 2.3.1 Screening for milk and cream fermentation

Twelve LAB isolates with unique morphologies were selected, streaked onto MRS agar, and incubated at 37 °C for 36 h. For each isolate, a single isolated colony was inoculated into 10 mL of MRS broth and incubated at 37 °C for 18 h with shaking at 200 rpm in a shaking incubator (MaxQ 6000, Thermo Fisher Scientific). After incubation, cultures were harvested by centrifugation (9,615 × *g* for 10 min), washed and resuspended in 1 mL of sterile phosphate-buffered saline (PBS, Fisher Scientific). Aliquots (100 μL) of each resuspended LAB isolate were inoculated into 10 mL of pasteurized whole milk (Kroger, Cincinnati, Ohio, USA). Similarly, a mixture of all LAB isolates was used to inoculate milk samples. The milk samples inoculated with individual LAB isolates or a mixture of all isolates were incubated at 37 °C for 24 h, and the coagulation status of milk was recorded every 2 h for 12 h. LAB isolates that failed to coagulate whole milk within 8 h were excluded from downstream testing.

Following the exclusion of LAB isolates unable to ferment and coagulate whole milk within 8 h, five isolates demonstrated high coagulation potential of milk within 8 h. These five LAB isolates were used to inoculate 10 mL MRS broth and incubated at 37 °C for 18 h with shaking at 200 rpm in a shaking incubator. The LAB cultures were harvested by centrifugation (9,615 × *g* for 10 min at 4 °C), washed, and resuspended in 1 mL of PBS. Aliquots (100 μL) of the cell suspension of each isolate were used to inoculate 10 mL of pasteurized whole milk or pasteurized heavy cream (Kroger, Cincinnati, Ohio, USA). In addition, 100 μL of each of the five isolates was combined, vortexed, and 100 μL of that LAB mixture of isolates was used to inoculate 10 mL of pasteurized whole milk or pasteurized heavy cream. Control pasteurized whole milk or heavy cream was inoculated with 100 μL PBS instead. The inoculated milk or heavy cream samples were incubated at 37 °C for 8 h for milk and 24 h for cream. The coagulation and pH of these dairy products were assessed every 2 h for 12 h for fermented milk or for 24 h for cream samples. Populations of LAB were determined before and after coagulation of the fermented milk and cream samples by plating on MRS agar and incubating at 37 °C for 36 h. Coagulated milk and cream products were stored at 4 °C for downstream applications.

#### 2.3.2 Genomic analysis of the lactic acid bacteria strains

DNA was extracted from the five LAB strains that fermented (i.e., coagulated) milk and cream. The selected isolates were cultured in MRS broth for 24 h at 37 °C with shaking at 200 rpm in a shaking incubator, and the genomic DNA was extracted from 1.5 mL culture using a genomic DNA extraction kit (Qiagen QIAamp DNA Mini Kit, Qiagen) as described by the manufacturer. The concentration of the genomic DNA was measured using a fluorometer (Qubit 4, ThermoFisher Scientific). DNA libraries of the five strains were prepared, indexed, and normalized as described in a previous section (Section 2.2). The genomic DNA libraries were sequenced using AVITI sequencing (Element Biosciences) as described earlier (Section 2.2). Quality checks of raw reads were performed with FastQC (v0.12.1), and adaptors were trimmed with Trimmomatic (v.36). The raw reads were assembled using SPAdes genome assembler (v4.2.0), followed by assessment of genome completeness using CheckM (v1.0.18). The genome taxonomy database-toolkit (GTDB-Tk, v2.3.2) was used for taxonomic classification, and the JSpeciesWS server (v5.0.3) was used to calculate the isolates' average nucleotide identity (ANI) to assess any potential duplicate isolates.

#### 2.3.3 Protein determination of the fermented milk and cream products

A protein analyzer (LECO, St. Joseph, Michigan, USA) was used to determine the protein content of the fermented milk and cream products. Uninoculated whole milk (a food matrix control) was tested through the protein analyzer to ensure its proper function. A standard curve consisting of five varying EDTA concentrations (LECO, St. Joseph, Michigan, USA) was established, with an *R*^2^ value of 0.999. For each fermented product, a weighed portion of homogenized fermented milk or cream was transferred into tin capsules (LECO) in triplicate, and total nitrogen content was determined. Protein content was calculated automatically by the instrument from total nitrogen using a nitrogen-to-protein conversion (Jones) factor of 6.38.

#### 2.3.4 Moisture content determination of the fermented milk and cream products

The moisture analysis protocol was adapted from AOAC Official Method 990.20. The coagulated milk and cream products were removed from refrigeration and brought to room temperature. Then, the fermented products (3 g each) were weighed into pre-weighed aluminum pans, which were then incubated in a drying oven for four hours at 100 °C. After drying, the pans were placed in a desiccator and allowed to cool for 45 min. Dried coagulation products and pans were weighed to determine total mass. Total solids percentage was determined by the following:


$$\begin{array}{l} Total\;Solids\;Percentage\;=\\ \frac{\left(Mass\;of\;crucible\;with\;dried\;product-Mass\;of\;crucible\right)}{Mass\;of\;the\;fermented\;product\;before\;drying}*100 \end{array}$$

To convert to total moisture content, the following conversion was used:


$$\begin{array}{l} Moisture\;Content\;Percentage=100-Total\;solids\;percentage \end{array}$$

#### 2.3.5 Fatty acid profiling of the fermented dairy products

Methods for fatty acids profiling were adapted from a previous study (Lock et al., 2013) and performed in the dairy lipids Laboratory at Michigan State University. In fatty acid profiling, fermented milk samples were first freeze-dried, while fermented cream samples were centrifuged at 17,000 × *g* for 30 minutes at 4 °C to form a fat cake. Samples were treated with an adapted protocol from Lock et al. (2013) and Hara and Radin (1978) using n-hexane/isopropanol (3:2, vol/vol) for the extraction of total lipids from fat cakes. Fatty acid methyl esters (FAME) were extracted through the addition of 2.5 mL of n-hexane, 25 mg of lipids, and 0.5 mL of 0.5 M sodium methoxide, combined in methanol and shaken for 5 min. One gram of sodium bisulfate was added to the mixture and vortexed for mixing. Mixtures were then centrifuged at 6,000 × *g* for 5 min, with the FAME-containing supernatant being transferred to 2-mL vials for direct Gas Chromatographic (GC) analysis. Fatty acid compositional analysis in the C4:0 to C24:0 range was determined through a GC-2010 Plus gas chromatograph (Shimadzu, Kyoto, Japan) with split injection (1:100 split ratio) and a flame-ionization detector (FID) and a CP-Sil 88 WCOT fused-silica column (100 m × 0.25 mm x 0.2-μm film thickness, Varian Inc., Lake Forest, CA). Hydrogen was utilized as a carrier with a flow rate of 1 mL/min and 40 mL/min for the FID. The FID gas composition also contained purified air at 400 mL/min and nitrogen makeup gas at 30 mL/min. The temperatures of the detector and the injector were kept constant at 250 °C. For the oven program, an initial temperature of 40 °C was held for 4 min, then ramped at 13 °C/min to 175 °C and held for 27 min and finally ramped at 4 °C/min to 215 °C and held for 35 min. An injection volume of 1 μL was utilized.

Identification and quantification of peaks were performed using GC software (GCsolution software version 2.32.00; Shimadzu). Individual FAME were identified via comparisons to known retention times with FAME standards (GLC reference standard 463, GLC reference standard 481-B, and conjugated octadecadienoic mixture #UC-59-M from Nu-Chek Prep Inc., Elysian, MN; Supelco 37 component FAME mix, cis/trans FAME mix, bacterial acid methyl ester mix, and PUFA No. 3 mix from Supelco Inc., Bellefonte, PA). Short-chain FAMEs were corrected via response factors through previously published factors by Ulberth and Schrammel (1995). Concentrations of individual FA were expressed as g/100 g total fats of the fermented product (Schauff and Clark, 1992; Glasser et al., 2007). For the fatty acid composition analysis, fatty acids were grouped into short-chain fatty acids (SCFAs; C1-C5), medium-chain fatty acids (MCFAs; C6-C12), and long-chain fatty acids (LCFAs; ≥C13) (Layden et al., 2013; Vacca et al., 2026). The group sum obtained from each isolate was calculated by summing individual fatty acids belonging to each category. To assess fermentation-associated changes, fatty acids group sums were reported as net changes relative to the uninoculated control milk or cream controls.

### 2.4 Statistical analysis

All statistical analyses and graphical illustrations of the results were performed using GraphPad Prism (v 10.6.1). Experiments were performed in triplicate, and statistical analyses using the appropriate methods (One-way ANOVA or Šídák's multiple-comparison) were performed as mentioned in the figure legends. The *p*-value of 0.05 was the threshold below which lower values were considered significant. Different significance illustrations are designed as follows: ns: *p* > 0.05, ^*^: 0.01 ≤ *p* < 0.05, ^**^: 0.001 ≤ *p* < 0.01, ^***^: 0.0001 ≤ *p* < 0.001, ^****^: *p* < 0.0001.

## 3 Results

Five artisanal cheeses were analyzed by shotgun metagenomic sequencing. Their microbial community structure and metagenome-assembled genomes (MAGs) were reconstructed and functionally annotated from the metagenomic reads. In parallel with the bioinformatic analyses, five LAB strains were isolated from some of these cheeses, their whole genomes were sequenced, and their performance in fermenting milk and cream was evaluated.

### 3.1 Cheese metagenomes show a high abundance of diverse lactic acid bacteria and fungal species

**Figure 2:**
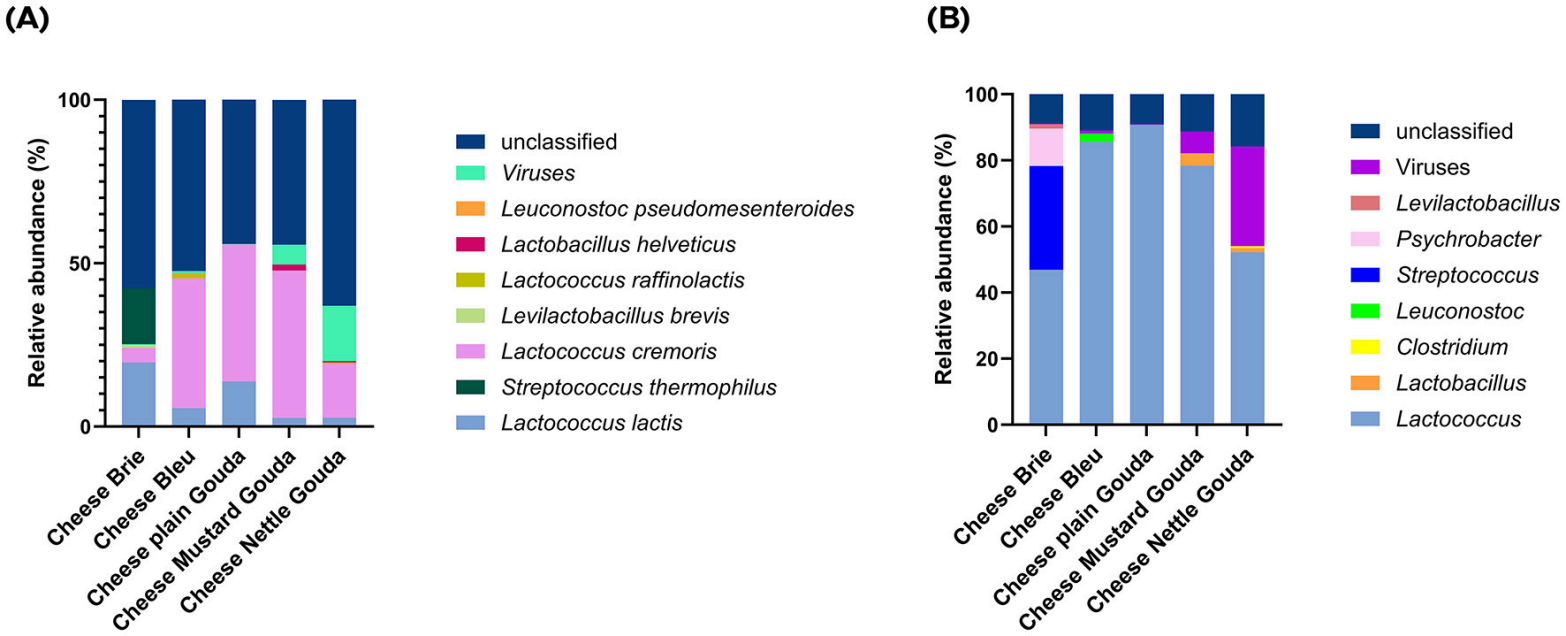
Taxonomic profiling of bacteria at the **(A)** species level and **(B)** genus level, expressed as relative bacterial abundance in the cheeses based on the shotgun metagenomic reads. Each column represents a cheese metagenome, and each color indicates the abundance of different bacterial **(A)** species or **(B)** genus.

Despite the high percentage of unclassified reads in the bacterial species abundance profile (Figure 2A), the *Lactococcus* genus dominated at the genus level (Figure 2B). In addition, different LAB species dominated the classified bacterial taxa. Among the five cheeses, *Lactococcus cremoris* (*L. cremoris*) and *Lactococcus lactis* (*L. lactis*) were the core LAB. *Lactococcus cremoris* had the highest percentage in all cheeses except Brie, which had higher percentages of *L. lactis* and *Streptococcus thermophilus*. Variations in bacterial taxa compositions were indicative of cheese production processes with the raw milk cheeses demonstrating core bacterial community, but the brie cheese produced with commercial strains and raw milk demonstrating a varied community composition. Overall, bacterial community dynamics, explored in cheese metagenomes, demonstrated core bacterial community structures consisting of a few key species.

**Figure 3:**
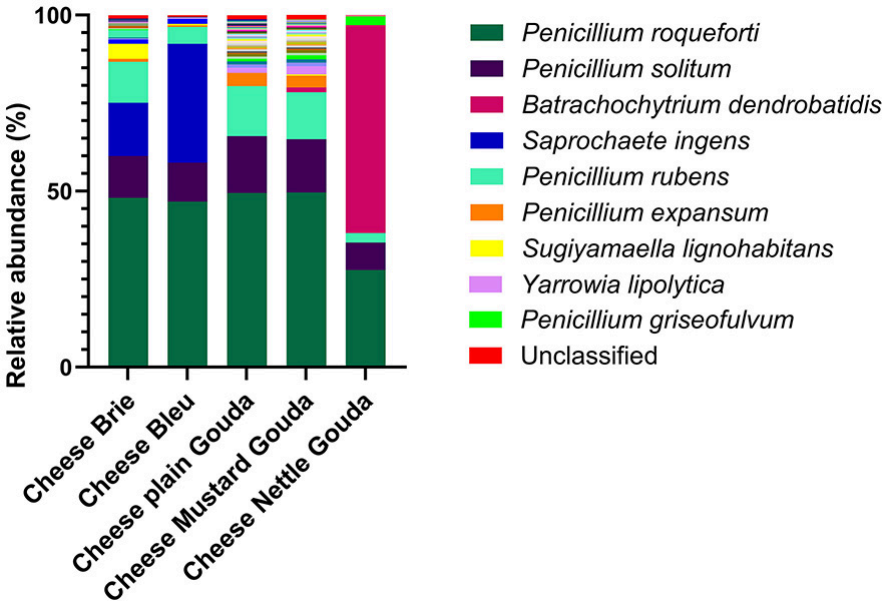
Taxonomic assessment of fungi at the species level, expressed as relative fungal abundance in the cheeses based on the shotgun metagenomic reads. Each column represents a cheese metagenome, and each color indicates the abundance of different fungal species. The top 10 abundant bacterial species were labeled as shown in the figure legend.

For fungi (Figure 3), *Penicillium roqueforti* and *Penicillium solitum* were the core fungal species among the cheeses. *Penicillium roqueforti* had the highest abundance in most cheeses. However, *Batrachochytrium dendrobatidis* had the highest abundance in Nettle Gouda cheese. Additional fungal species such as *Penicillium solitum, Saprochete ingens*, and *Penicillium rubens* also had significant abundance percentages among all cheeses. Such variations in fungal abundances are cheese-type dependent and are directly associated with the properties of the cheese types and methods of manufacturing. Brie and bleu cheeses had high relative abundances of known fungal populations (e.g., *Penicillium* species and *Saprochaete ingens*) due to their higher moisture content, along with production methods that favor the addition of starter fungi for flavor and sensory contributions. Interestingly, Gouda cheeses demonstrated high fungal diversity but lower relative abundance of certain taxa, such as *Saprochaete ingens*, compared to Brie and Bleu cheeses. This pattern may reflect differences in cheese-making and ripening conditions.

### 3.2 Cheese metagenome-assembled genomes encompass diverse lactic acid bacteria

**Figure 4:**
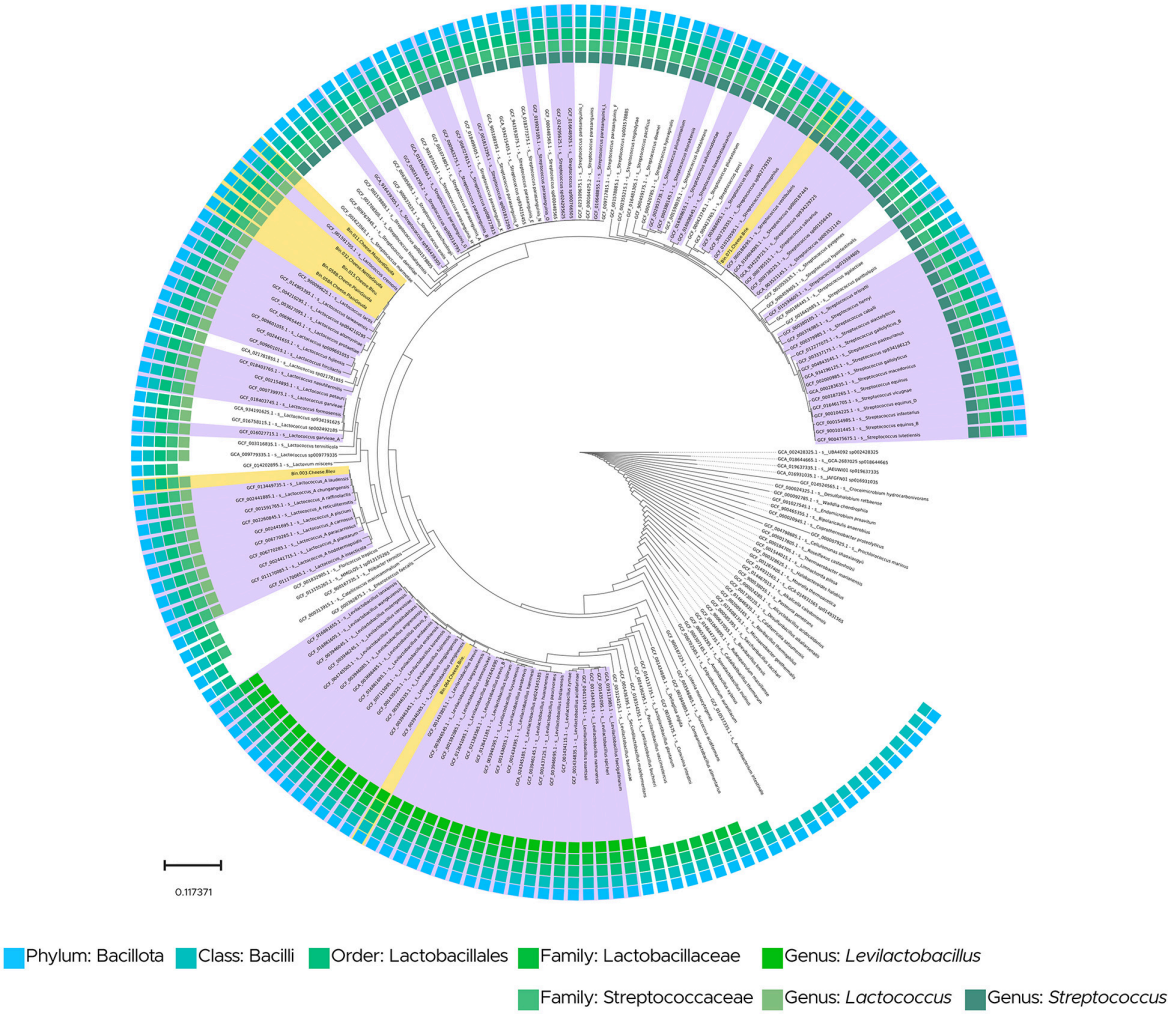
Phylogenetic analysis of the metagenome-assembled genomes (MAGs) using genome taxonomy database tools. Each MAG, is labeled by its bin identifier and cheese type of origin. The MAGs are highlighted in yellow and classified with the closely related genomes in the phylogenetic tree.

**Figure 5:**
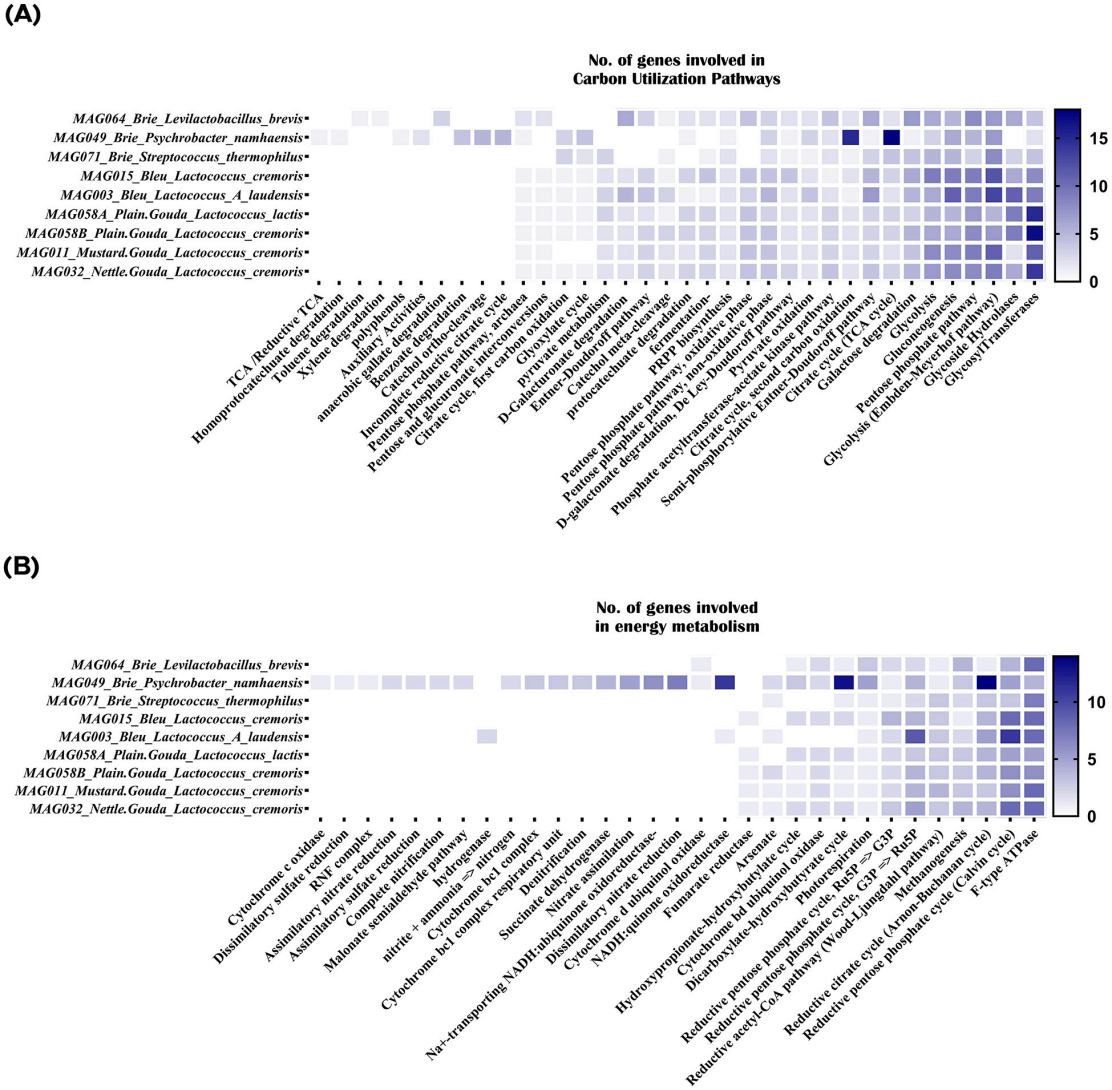
Heatmaps showing number of genes involved in **(A)** carbon utilization pathways and **(B)** energy metabolism among the metagenome-assembled genomes (MAGs). Each row represents a MAG, labeled by its bin identifier and the cheese type of origin. Darker shades indicate a greater number of genes, while lighter shades indicate a lower number of genes, as illustrated in the heatmap scale. Gene annotations were analyzed by DRAM and illustrated by GraphPad Prism.

**Figure 6:**
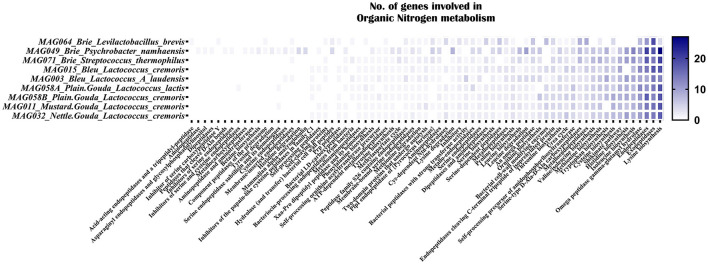
Heatmap showing the number of genes involved in organic nitrogen metabolism among the metagenome-assembled genomes. Each row represents a MAG, labeled by its bin identifier and the cheese type of origin. Darker shades indicate a greater number of genes, while lighter shades indicate a lower number of genes, as illustrated in the heatmap scale. Data were obtained from DRAM and illustrated by GraphPad Prism.

The metagenome-assembled genomes (MAGs; expressed as binned contigs in Figures 4–6) derived from the cheese metagenomes spanned a wide range of lactic acid bacteria. Brie cheese had MAGs comprising *Psychrobacter namhaensis, Levilactobacillus brevis*, and *Streptococcus thermophilus*, while Bleu cheese MAGs included *Lactococcus laudensis* and *L. cremoris*. Additionally, MAGs from Plain Gouda cheese included *L. lactis* and *L. cremoris*, while cheese nettle gouda MAGs included *L. cremoris* only.

### 3.3 Cheese metagenome-assembled genomes have a high abundance of F-type ATPases and glycosyl transferases

The MAGs were mined for functional pathways to gain insight into the key metabolic pathways related to their ability to coagulate and ferment milk and cream. In Figure 5A, carbon utilization pathways such as glycolysis, gluconeogenesis, and galactose metabolism are enriched in all cheeses. Additionally, all cheeses had a high number of glycosyl transferase and glycoside hydrolase genes, with Brie cheese MAGs showing a relatively lower number of carbon utilization genes than other cheeses. However, Brie cheese MAGs were enriched in pathways that were absent in other cheeses, such as catechol-ortho cleavage, and degradation of benzoate, anaerobic gallate, polyphenols, toluene, and xylene.

In Figure 5B, most cheeses' MAGs were enriched in essential genes for energy metabolism, such as F-type ATPases and genes involved in the reductive pentose phosphate cycle, decarboxylate-hydroxybutyrate cycle, and the reductive citrate cycle. Interestingly, one MAG (MAG049_Brie_*Psychrobacter_namhaensis*) in Figure 5B was enriched in many pathways involved in energy metabolism.

The MAGs were also mined for pathways involved in organic nitrogen metabolism (Figure 6). Most of the MAGs contained genes for amino acid biosynthesis, including lysine, isoleucine, proline, and serine. Also, they had different types of peptidases and aminotransferases. A MAG (MAG049_Brie_*Psychrobacter_namhaensis*) from brie cheese was enriched in specific types of peptidases like metallocarboxypeptidases and asparaginyl endopeptidases.

### 3.4 The selected cheese LAB strains were classified as *Lactococcus lactis*

The five isolated LAB strains were genomically classified using genome taxonomy database toolkit and belonged to the *L. lactis* species and their accessions numbers are provided in Table 1. The isolates had an ANI range of 97.40–99.96 to each other and a coverage range of 79.40–96.10, indicating there is no duplicate isolates.

**Table 1:** Accession numbers of the cheese metagenomes and whole genome sequences of the five isolated *L. lactis* strains.

Sample	Biosample accession no.	SRA accession no.	Genome accession no.
Cheese_Brie_metagenome	SAMN53701434	SRR36940308	N/A
Cheese_Bleu_metagenome	SAMN53701435	SRR36940307	N/A
Cheese_Plain_Gouda_metagenome	SAMN53701436	SRR36940306	N/A
Cheese_Mustard_Gouda_metagenome	SAMN53701437	SRR36940305	N/A
Cheese_Nettle_Gouda_metagenome	SAMN53701438	SRR36940304	N/A
*L.lactis*_BC-1	SAMN53701467	SRR36940303	JBTYZT000000000
*L.lactis*_BC-2	SAMN53701468	SRR36940302	JBTYZS000000000
*L.lactis*_BC-3	SAMN53701469	SRR36940301	JBTYZR000000000
*L.lactis*_NGC-1	SAMN53701470	SRR36940300	JBTYZQ000000000
*L.lactis*_NGC-2	SAMN53701471	SRR36940299	JBTYZP000000000

### 3.5 The *L. lactis* strains fermented and coagulated milk and cream

**Figure 7:**
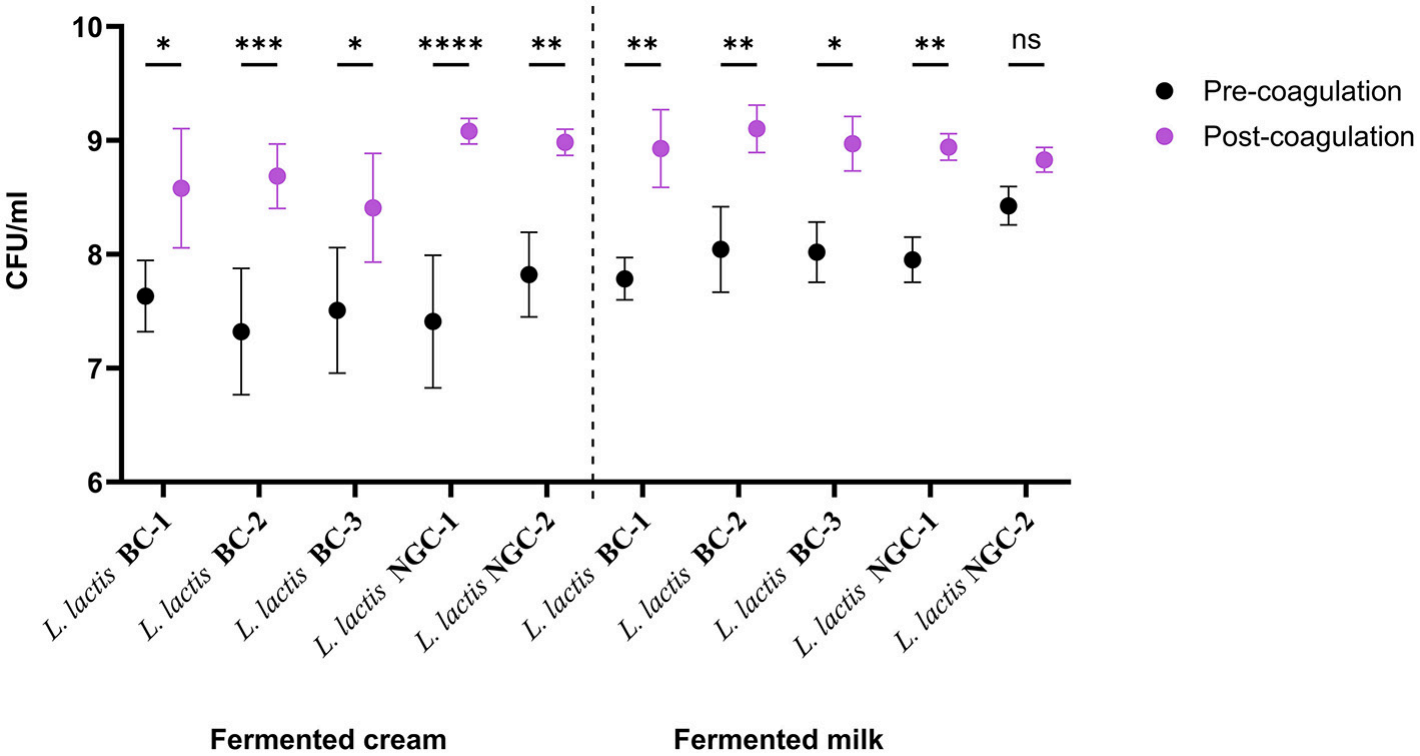
Populations (CFU/ml) of *L. Lactis* strains pre- and post-coagulation of fermented cream **(left)** and milk **(right)**. Black circles indicate colony counts pre-coagulation, and purple circles indicate post-coagulation, as illustrated in the figure legend. Significance was determined using Šídák's multiple-comparison test after a two-way ANOVA test analysis. Significance is read as follows: ns: *p* > 0.05, *0.01 ≤ *p* < 0.05, **0.001 ≤ *p* < 0.01, ***0.0001 ≤ *p* < 0.001, ****p < 0.0001.

**Figure 8:**
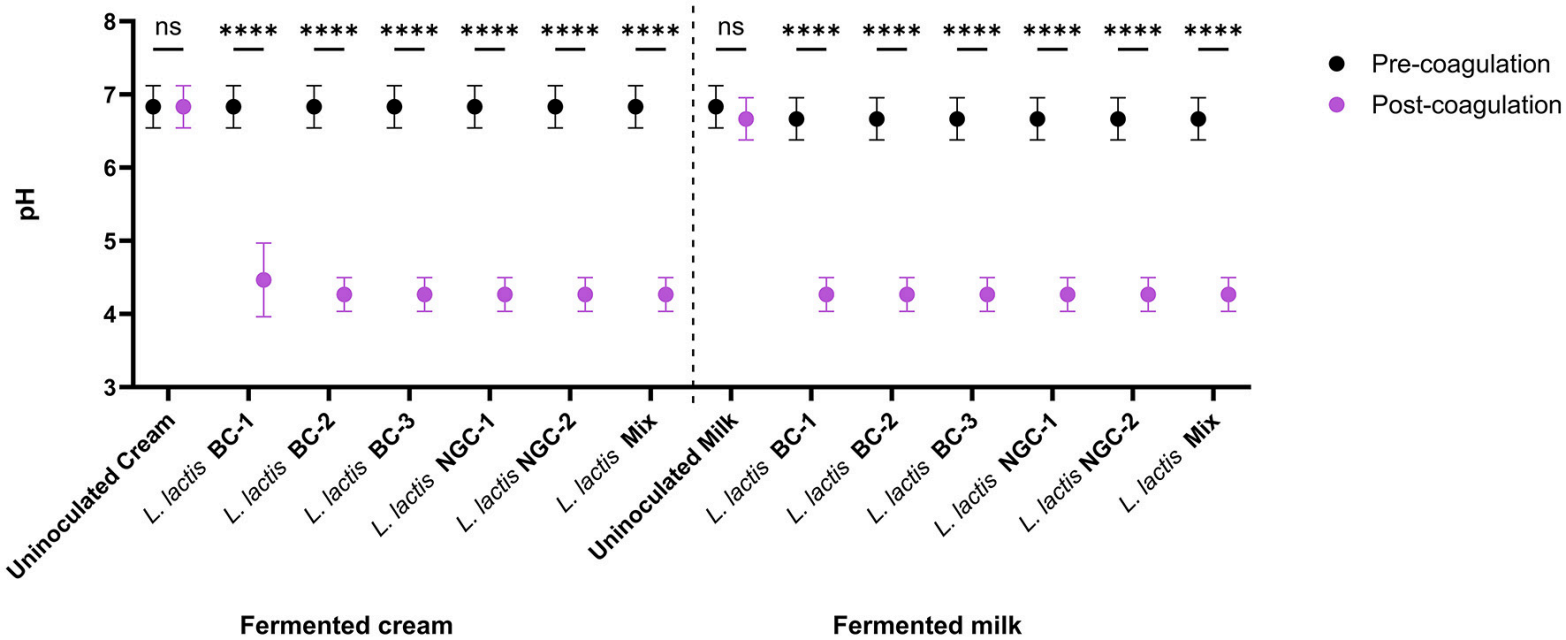
The pH of fermented dairy products pre- and post-coagulation in sour cream **(left)** and milk **(right)**. Black circles indicate pH pre-coagulation, and purple circles indicate post-coagulation, as illustrated in the figure legend. Significance was determined using Šídák's multiple-comparison test after a two-way ANOVA test analysis. Significance is read as follows: ns: *p* > 0.05, ****p < 0.0001.

The five *L. lactis* strains were able to grow and ferment both dairy products and displayed a significant increase (*p* < 0.05) in populations (Figure 7) after coagulation of both milk and cream, despite having only one strain (*L. lactis* NGC-2) with non-significant growth in milk (Figure 7). In addition, pH decreased drastically (*p* < 0.05) in all coagulated dairy products (Figure 8).

### 3.6 Chemical properties of the fermented milk and cream products using *L. lactis* strains

**Figure 9:**
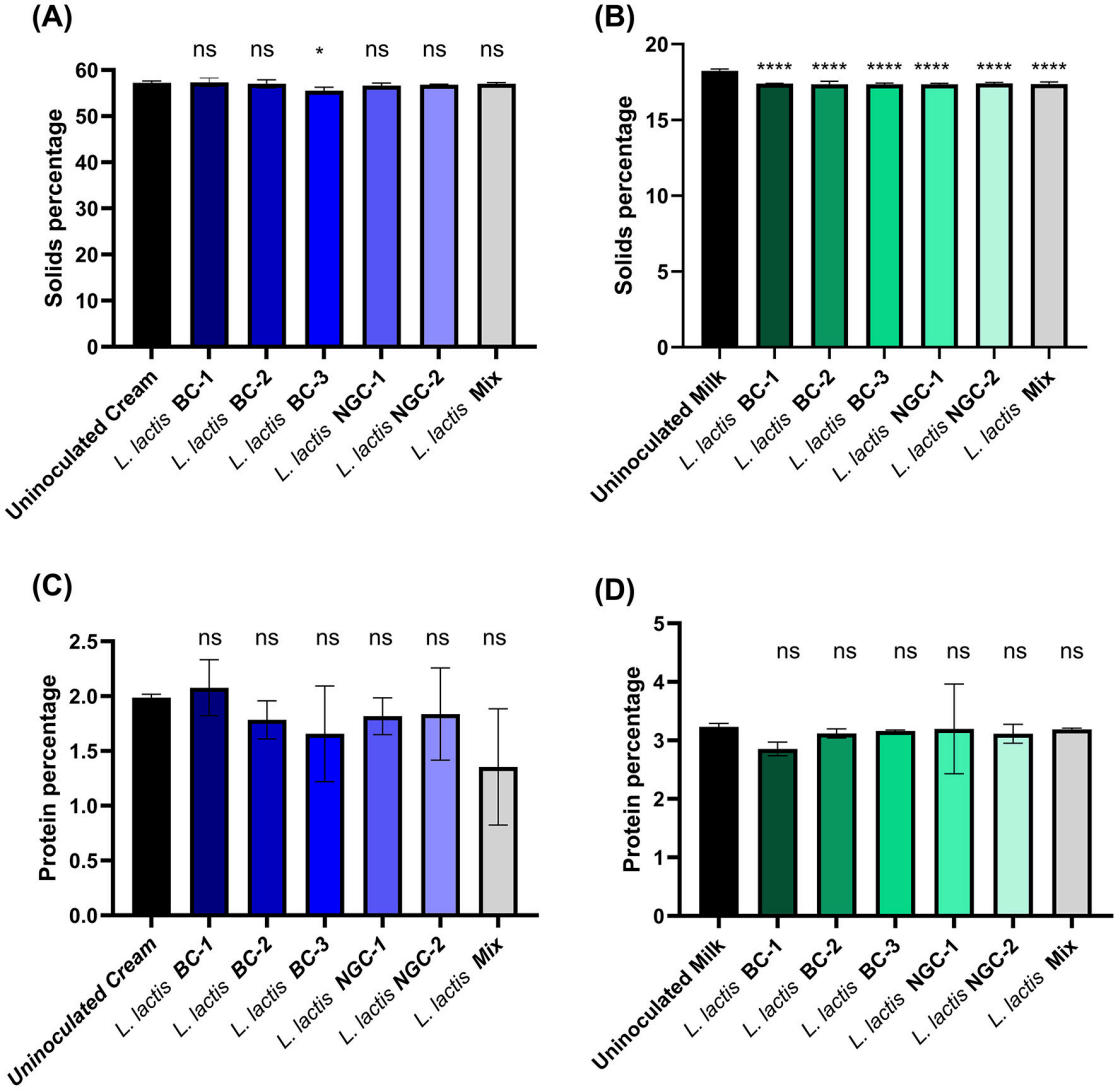
Total solids percentage (**A,B**) and protein percentage (**C,D**) of fermented cream (blue bars) and fermented milk (green bars) by different strains of *L. lactis*. Significance was determined using an ordinary one-way ANOVA test. Multiple comparisons significance is read as follows: ns: *p* >0.05, *0.01 ≤ *p* < 0.05, ****p < 0.0001.

The total solids percentage of fermented dairy products was determined and compared with that of uninoculated products. The total solids in fermented cream were insignificantly different (*p* > 0.05) among all isolates except *L. lactis* BC-3 (Figure 9A). However, the total solids decreased slightly, but with a significant decrease across all the isolates in fermented milk (Figure 9B). The protein percentage of fermented cream (Figure 9C) and fermented milk (Figure 9D) showed no significant changes compared to uninoculated milk or cream controls. The protein content of the fermented products ranged from 2.9% to 4.2%.

**Figure 10:**
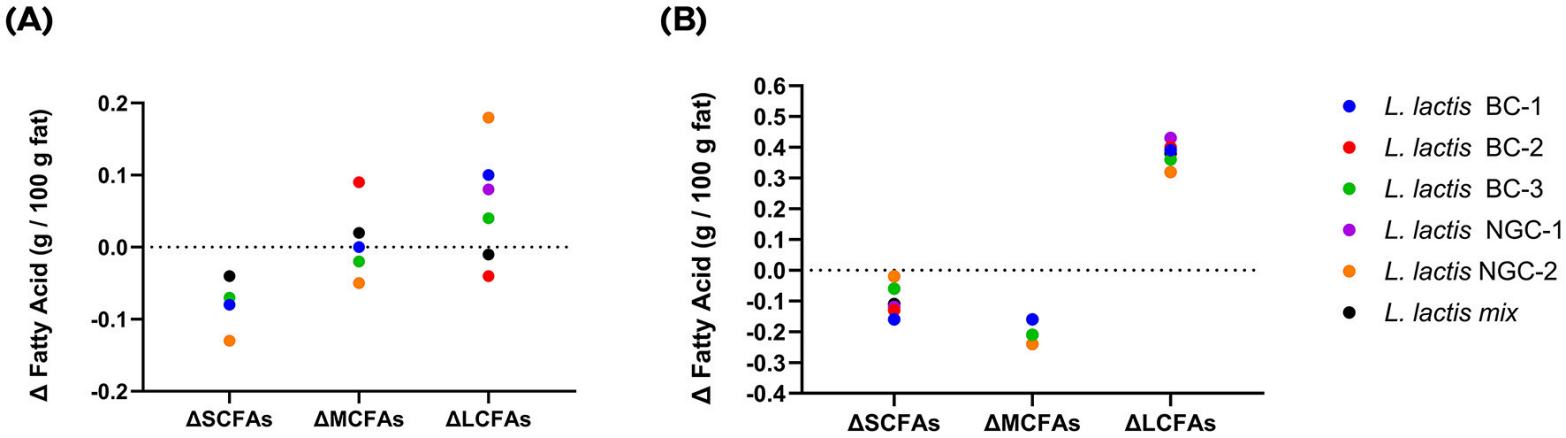
Changes in fatty acid classes in fermented cream and milk using different strains of *L. lactis* relative to un-inoculated controls. **(A)** Cream and **(B)** Milk. SCFAs: short-chain fatty acids, MCFAs: medium-chain fatty acids, and LCFAs: long-chain fatty acids.

When observing the fatty acid profiles of the fermented products (Figure 10), SCFAs showed a relative decrease in comparison to uninoculated controls in both milk and cream. MCFAs had minimal changes in fermented cream (Figure 10A), in contrast to a greater decrease in fermented milk (Figure 10B). On the other hand, LCFAs showed a relative increase in both fermented milk and cream products, with a greater increase in the fermented milk. Concentrations of individual fatty acids are provided in Supplementary file 2.

## 4 Discussion

In commercially produced fermented dairy products, the utilization of starter cultures is paramount for consistent quality of the products (González-González et al., 2022). However, given the current lack of variety of starter cultures in fermented dairy products and the consumer requirement for nutritional, sensorial, and probiotic attributes of dairy products, the need for new strains of starter cultures in this industry is strong (Popović et al., 2020). Thus, raw milk cheeses produced by artisanal methods were analyzed in this study for their capacity to serve as reservoirs for novel LAB isolates for potential use in dairy production.

Metagenomic analyses were performed to understand how bacterial communities behave in cheese environments. By assessing the taxonomic abundances of the five artisanal cheeses studied, low diversity among bacterial species was observed (Figure 2A); however, taxonomic assignment improved substantially at the genus level (Figure 2B). The limited representation of artisanal cheese-associated strains in reference databases could introduce difficulty in resolving closely related LAB species from the short metagenomic reads. Nonetheless, significant LAB strains for dairy production were identified, including *L. lactis* and *L. cremoris*. Additionally, given the high diversity within the fungal microbiome (Figure 3), there is a strong indication that many of the fungal isolates would be unable to be cultured in a laboratory setting due to symbiotic relationships that are typically observed in similar diverse taxonomic structures (Zhang et al., 2018). Additionally, by examining the complex microbiota of raw milk cheeses, it is likely that fungal-bacterial interactions occur, allowing the creation of desirable sensorial and nutritional characteristics that would not typically be observed in cheeses ripened by only a bacterial starter culture.

Based on phylogenetic analysis of the cheese microbiome (Figure 4), most cheeses harbor *L. cremoris* strains, indicating their functional importance in the cheese microbiome. *L. cremoris* is known to regulate volatiles like diacetyl and acetoin that lead to the cheese's buttery flavor (Melkonian et al., 2023). Additionally, the metagenomic analysis of cheeses yielded MAGs belonging to other species, such as *P. namhaensis, L. brevis, L. laudensis*, and *L. lactis*, which functionally complement the microbial community through flavor development, hetero-fermentation, and aroma production (Tamang et al., 2016; van Wyk, 2024). Such distinct profiles of LAB enable complex metabolic pathways, and complement the fermentation processes they undergo as a community. This assumption is supported by findings that many of the LAB isolates contained genes encoding amino acid synthesis and F-type ATPases, which typically allow the continuation of fermentative conditions under acidic stress (Figure 5) (Gevorgyan and Trchounian, 2025). The presence of such ATPases enables intracellular homeostasis under acidic conditions, a critical necessity for LAB, which can lower pH to approximately 4.5 (Bang et al., 2014). This notion is also supported by the high abundance of glycosyl transferases and glycoside hydrolases (Figure 5), indicating that these MAGs in the cheese microbiome have high fermentation potential and ability to break down sugars during fermentation to sustain fermentative growth (Chang et al., 2025). Additionally, significant metabolic differences of MAGs were observed between cheeses, specifically between Brie and the Gouda variants (Figures 5 and 6), indicating that the cheese environment and production methods impact the composition and metabolism of the naturally occurring microbiota and, thus, affect the quality attributes of the end cheese product (Reuben et al., 2023).

When analyzing the metagenomic profile for each of the cheeses in the current study, there is a high proportion of unclassified reads at the bacterial species level (Figure 2A) and a lower number of extracted MAGs in comparison to the high levels of microbial diversity observed in the taxonomic abundance of all five cheeses. This could be due to several assumptions, including limitations in the prokaryotic reference genome databases (Almeida et al., 2019), the presence of low-abundance taxa (Sczyrba et al., 2017), and the stringent quality thresholds (e.g., 90% completeness of the genomes) that have been implemented to extract MAGs (Li and Yin, 2022).

Following cheese microbiome analyses, strains of only *L. lactis* were isolated through the laboratory methods used in the current study. However, as observed in the metagenomic data, there were metagenomic reads indicating greater diversity among the existing bacterial taxa. The low number of cultured LAB isolates from artisanal cheeses is a common trend, because the vast majority of metagenomically-identified taxa exist in complex microbial ecosystems and cannot be grown solely on a conventional microbiological medium (Widder et al., 2016). The complex nutritional relationships among the bacterial members of a food microbiome pose a significant challenge against culturability, explaining why only a subset of bacterial species can be isolated from artisanal cheese sources.

Using laboratory methods, the isolated LAB strains were evaluated in small-scale tests to assess their potential as starter cultures to ferment dairy products. This is important for determining which strains are worthy of study, because starter cultures in the dairy industry need to be both safe and effective at producing fermentation products in a short time frame to maximize profit efficiency for industry. The isolated LAB strains, in this study, belonged to *L. lactis*, and this species is known to carry a wide array of genes for sugar metabolism, aminopeptidases that contribute to flavor formation, and amino acid biosynthesis and degradation genes that help in aroma production and coagulation of milk casein (Malekijahan et al., 2025). This was obvious when the five strains of *L. lactis* fermented and coagulated milk and cream in the current study (Figure 7). Additionally, through F-type ATPase genes, the isolated *L. lactis* strains can withstand the high acidity of fermented dairy products and support their continued growth to pH ranges about 4.5 (Figure 8). This LAB growth in acidic environment is demonstrated by the noticeable increase in the populations of all LAB strains of *L. lactis* (Figure 7) in fermented milk and cream products. This pattern is a crucial indicator for the ability of such LAB strains to serve as potential starter cultures in the fermented dairy space.

In addition to screening *L. lactis* isolates for their capacity to serve as starter cultures for fermenting milk and heavy cream products, it was important to characterize the nutritional properties of the fermented products produced by such isolates. This is important because consumers and producers of fermented dairy products demand products that are both appealing to taste and good for their health (Jakubowska et al., 2024). Thus, this rationale highlights the importance of testing fermented dairy products for their nutritional properties. Specifically, by analyzing the percentage of total solids of the fermented milk and cream samples, it was noted that there were no significant differences between the *L. lactis* strains (Figure 9). However, for both the fermented milk and cream samples, the percentages of total solids values were in line with prior studies (Yadav et al., 2018; Shepard et al., 2013), indicating a high degree of consistency of the end dairy product. Additionally, the protein content of the fermented milk and cream samples produced by the *L. lactis* strains shows a slight trend toward a decrease in protein content compared to uninoculated milk or cream (Figure 9). This observation is supported by the metabolic processes and proteolytic activities that these LAB strains exhibit during fermentation (Harper et al., 2022).

It is well known that in sour cream products, variations in starter culture consortium result in significant variations in the FA profiles, along with other sensorial and textural changes (Khademi et al., 2022). Overall, LAB possess the capacity to produce folates, B vitamins, and cis-9, trans-11 conjugated linoleic acid C18:2 (CLA) during cold storage in produced dairy products (Czarnowska-Kujawska and Paszczyk, 2021). CLA has been associated with anti-inflammatory, antioxidant, and anti-carcinogenic activities (Badawy et al., 2023). By observing the FA profiles of the fermented milk and cream in the current study, the fermented products showed varied changes in CLA relative to controls; however, overall values were lower than those of typical commercially produced yogurt products (Serafeimidou et al., 2012). Further research is needed to establish such CLA and LAB associations. Additionally, SCFAs showed minimal decreases relative to controls, reflecting the dynamic changes in the FA profile associated with fermentation (Baxter et al., 2019). On the other hand, LCFAs increased (Figure 10), including specific FAs associated with dairy products, such as an increase of C16:0 (Palmitic Acid) in milk, C18:0 (Stearic Acid) in both cream and milk, and C18:1 9c in milk (Supplementary file 2). The fermented milk products showed a greater increase in LCFAs than fermented cream products, indicating that different food matrices have varied effects on fermentation end products (Huppertz et al., 2024).

Overall, the five novel LAB strains isolated in the current study, can ferment and coagulate milk and heavy cream dairy products, with desirable physicochemical properties (total solids, protein content, moisture, and fatty acids) that indicate that the strains could be potential starter cultures in large scale application that enhance the sensory and textural components of the fermented dairy products.

## 5 Conclusion

For the isolation and characterization of new LAB strains from raw milk and artisanal cheeses, there are a few critical considerations. Firstly, raw milk cheeses serve as diverse reservoirs for novel LAB, allowing the isolation of new strains that add value to the commercial fermented dairy industry. Secondly, the distinct microbiota of raw milk artisanal cheeses supports the development of diverse sensory and textural characteristics that align with consumer preferences. Thirdly, for characterizing new LAB strains, pilot-scale dairy fermentation and sensorial analyses of the fermented products must be conducted to ensure that these strains produce end products that consumers require. In the current study, shotgun metagenomic profiling of artisanal cheeses indicated high abundance of *Lactococcus* species and five *L. lactis* strains were isolated from this small subset of cheeses. Thus, additional geographic locations and diverse dairy products should be further screened for potential new LAB strains with desirable phenotypic and genotypic traits. Additionally, future directions should explore the use of these LAB strains in novel food products, such as non-dairy fermented milk alternatives. In summary, artisanal cheeses can serve as valuable reservoirs of LAB suitable for starter application in the commercial dairy industry.

## Data Availability

The datasets presented in this study can be found in online repositories. The names of the repository/repositories and accession number(s) can be found in the article/Supplementary material.
