# Supplementary material for: Shotgun metagenomic and phenotypic characterization of indigenous lactic acid bacteria from raw milk artisanal cheeses: metagenomic functional insight and starter culture traits
Source: Front Microbiol. 2026 Jun 4;17:1820264. doi: 10.3389/fmicb.2026.1820264 (PMC13275648; doi:10.3389/fmicb.2026.1820264)
Supplement: Supplementary file 1 [file Table_1.docx]

**Supplementary Table 1: Assembly and MAGs quality metrics**

|  | Genome Size (bp) | N50 | Total No. of Contigs | No. of MAGs | CheckM Completeness | CheckM Contamination |
| --- | --- | --- | --- | --- | --- | --- |
| Cheese Brie | 95868211 | 27869 | 9990 | 3 | 96.68 | 6.37 |
|  |  |  |  |  | 99.06 | 0.63 |
|  |  |  |  |  | 98.13 | 0 |
| Cheese Bleu | 42797240 | 160825 | 1750 | 2 | 94.97 | 0.38 |
|  |  |  |  |  | 92.83 | 7.48 |
| Cheese Plain Gouda | 5374728 | 20426 | 482 | 2 | 92.94 | 4.81 |
|  |  |  |  |  | 90.89 | 0.27 |
| Cheese Mustard Gouda | 6821592 | 11874 | 863 | 1 | 98.7 | 0.75 |
| Cheese Nettle Gouda | 18541553 | 73053 | 1077 | 1 | 96.37 | 6.08 |
